# The FaceBase Consortium: a comprehensive resource for craniofacial researchers

**DOI:** 10.1242/dev.135434

**Published:** 2016-07-15

**Authors:** James F. Brinkley, Shannon Fisher, Matthew P. Harris, Greg Holmes, Joan E. Hooper, Ethylin Wang Jabs, Kenneth L. Jones, Carl Kesselman, Ophir D. Klein, Richard L. Maas, Mary L. Marazita, Licia Selleri, Richard A. Spritz, Harm van Bakel, Axel Visel, Trevor J. Williams, Joanna Wysocka, FaceBase Consortium, Yang Chai

**Affiliations:** 1 Structural Informatics Group, Department of Biological Structure, University of Washington, Seattle, WA 98195, USA; 2 Department of Pharmacology and Experimental Therapeutics, Boston University School of Medicine, Boston, MA 02118, USA; 3 Department of Orthopedic Research, Boston Children's Hospital and Department of Genetics, Harvard Medical School, Boston, MA 02115, USA; 4 Department of Genetics and Genomic Sciences, Icahn School of Medicine at Mount Sinai, New York, NY 10029, USA; 5 Cell and Developmental Biology, School of Medicine, University of Colorado, Aurora, CO 80045, USA; 6 Department of Biochemistry and Molecular Genetics, School of Medicine, University of Colorado, Aurora, CO 80045, USA; 7 Information Sciences Institute, Viterbi School of Engineering, University of Southern California, Marina del Rey, CA 90292, USA; 8 Program in Craniofacial Biology, Departments of Orofacial Sciences and Pediatrics, Institute for Human Genetics, University of California San Francisco, San Francisco, CA 94143, USA; 9 Division of Genetics, Brigham and Women's Hospital, Harvard Medical School, Boston, MA 02115, USA; 10 Center for Craniofacial and Dental Genetics, Department of Oral Biology, School of Dental Medicine, University of Pittsburgh, Pittsburgh, PA 15213, USA; 11 Department of Human Genetics, Graduate School of Public Health, University of Pittsburgh, Pittsburgh, PA 15213, USA; 12 Human Medical Genetics and Genomics Program, School of Medicine, University of Colorado, Aurora, CO 80045, USA; 13 Lawrence Berkeley National Laboratory, Berkeley, CA 94720, USA; 14 U.S. Department of Energy Joint Genome Institute, Walnut Creek, CA 94598, USA; 15 School of Natural Sciences, University of California Merced, Merced, CA 95343, USA; 16 Department of Craniofacial Biology, School of Dental Medicine, University of Colorado, Aurora, CO 80045, USA; 17 Department of Chemical and Systems Biology and of Developmental Biology, School of Medicine, Stanford University, Stanford, CA 94305, USA; 18 Center for Craniofacial Molecular Biology, Herman Ostrow School of Dentistry, University of Southern California, Los Angeles, CA 90033, USA

**Keywords:** Craniofacial development, Data resource, FaceBase Consortium

## Abstract

The FaceBase Consortium, funded by the National Institute of Dental and Craniofacial Research, National Institutes of Health, is designed to accelerate understanding of craniofacial developmental biology by generating comprehensive data resources to empower the research community, exploring high-throughput technology, fostering new scientific collaborations among researchers and human/computer interactions, facilitating hypothesis-driven research and translating science into improved health care to benefit patients. The resources generated by the FaceBase projects include a number of dynamic imaging modalities, genome-wide association studies, software tools for analyzing human facial abnormalities, detailed phenotyping, anatomical and molecular atlases, global and specific gene expression patterns, and transcriptional profiling over the course of embryonic and postnatal development in animal models and humans. The integrated data visualization tools, faceted search infrastructure, and curation provided by the FaceBase Hub offer flexible and intuitive ways to interact with these multidisciplinary data. In parallel, the datasets also offer unique opportunities for new collaborations and training for researchers coming into the field of craniofacial studies. Here, we highlight the focus of each spoke project and the integration of datasets contributed by the spokes to facilitate craniofacial research.

## Abstract (precis)

**Summary:** The FaceBase Consortium is a dynamic, comprehensive resource of craniofacial development, with hundreds of datasets that are freely available to the research community at the FaceBase Hub website.

## Introduction

Advances in biomedical research and bioinformatics coupled with the dramatic decrease in the cost of sequencing have contributed to a revolution in the quantity and variety of data that are available to researchers. At the same time, it has become apparent that improved understanding of developmental biology in health and disease depends on making connections between these different types of data and how they represent dynamic spatial and temporal changes during development. In 2009, the National Institute of Dental and Craniofacial Research (NIDCR) launched the FaceBase Consortium, designed to accelerate understanding of craniofacial developmental biology by generating comprehensive data resources to empower the research community, exploring high-throughput technology, fostering new scientific collaborations among researchers and human/computer interactions, facilitating hypothesis-driven research and translating science into improved health care to benefit patients.

In the first five years of FaceBase, the NIDCR provided funding support for eleven projects, selected through a peer-review process: a central coordinating center for data management and integration (‘the Hub’) and ten research and technology ‘spoke’ projects focused on the development of the midface. This first set of FaceBase endeavors included data generation as well as technology development and is detailed in Hochheiser et al. (2011). These efforts were successful in establishing atlases of several aspects of midface development in humans and animal models. The nearly 600 datasets generated by these projects are available at the Hub website, a publicly available resource provided to the scientific community to facilitate interaction with these datasets (facebase.org). To date, more than 100 publications have referenced these datasets. In parallel, the NIDCR has created opportunities for researchers to conduct secondary analyses of FaceBase datasets relevant to craniofacial development, human craniofacial conditions or traits and animal models of those craniofacial conditions (PAR-13-178); currently, three such projects have already been funded. Collectively, FaceBase has created a comprehensive resource for the craniofacial research community.

**Fig. 1.**
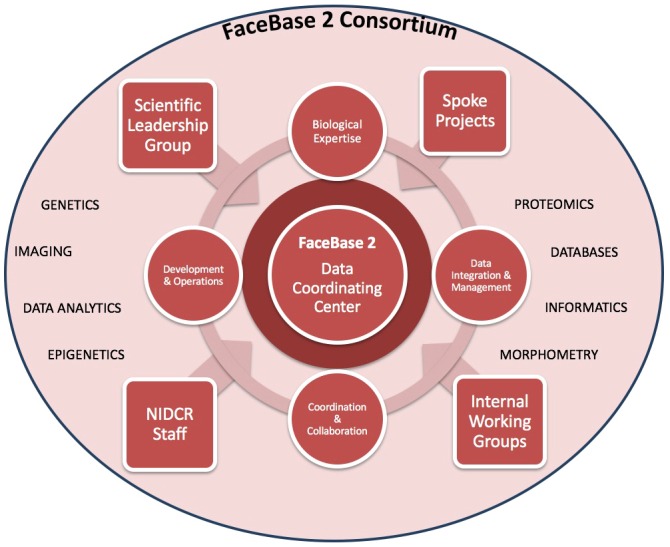
**Architecture of the FaceBase 2 Consortium.**

The second iteration of FaceBase (‘FaceBase 2’) was launched in 2014 with the funding of a new Hub and ten spoke projects (Fig. 1, Table 1). The scope of FaceBase was expanded at this time to anatomical structures of the craniofacial region beyond the midface and palate. The research objectives of FaceBase 2 remain highly relevant to the mission of the NIDCR, providing comprehensive datasets on craniofacial development that serve as freely available community resources. These datasets are designed to accelerate craniofacial research by providing a broad and deep pool of resources beyond what would normally be accessible to any one laboratory working independently. A number of projects include both human and animal models, providing a translational component; the ability to compare across several animal models also offers evolutionary perspectives.

**Table 1.**
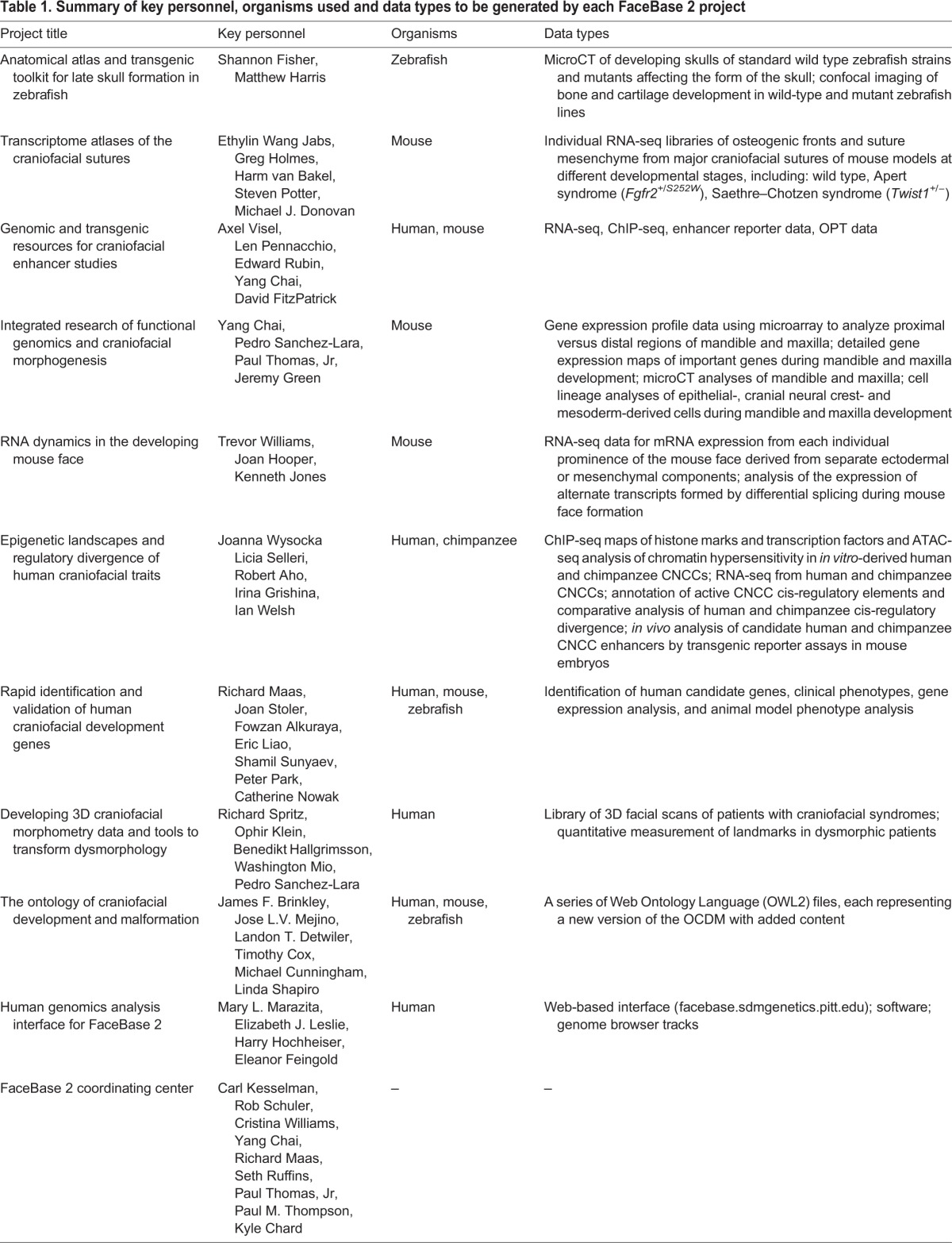
**Summary of key personnel, organisms used and data types to be generated by each FaceBase 2 project**

The resources generated by the FaceBase 1 and 2 projects include diverse types of data, such as a number of dynamic imaging modalities, genome-wide association studies, software tools for analyzing human facial abnormalities, detailed phenotyping, anatomical and molecular atlases, global and specific gene expression patterns, and transcriptional profiling over the course of embryonic and postnatal development in animal models and humans. The integrated data visualization tools, faceted search infrastructure and curation provided by the Hub offer flexible and intuitive ways to interact with these multidisciplinary data. In parallel, FaceBase datasets also offer unique opportunities for new collaborations and training for researchers coming into the field of craniofacial studies. Here, we highlight the focus of each spoke project and the comprehensive approach of the Hub to integrating datasets contributed by the spokes to facilitate craniofacial research.

## Data generated using animal models

### 

#### Anatomical atlas and transgenic toolkit for late skull formation in zebrafish

The final form of the adult skull is achieved through a complex series of morphogenetic events and growth, largely during post-embryonic development. Many common human congenital defects in the skull have their foundation in these developmental events. The treatment options in human patients are far from perfect and improvements demand a more complete understanding of the biology underlying post-embryonic skull formation. However, due to their complex development and relatively late occurrence, these clinically relevant stages in skull development have been less accessible in experimental organisms. The zebrafish displays fundamental similarity in skeletogenesis to mammals, including in formation of the vault of the skull and the cranial sutures (Cubbage and Mabee, 1996) and in developmental abnormalities (Fisher et al., 2003; Laue et al., 2011). Although the later events of skull and suture formation have been relatively less well studied in zebrafish, they are nonetheless accessible for manipulations and imaging, making the zebrafish an ideal system to further our understanding of these complex events (facebase.org/projects/anatomical-atlas-transgenic-toolkit-zebrafish/).

The overall aim of this project is to lay the foundation for the use of zebrafish to examine skull and suture formation. The first goal is to generate a comprehensive atlas of normal skull development, encompassing late larval to early adult stages during which the skull vault and sutures form. At earlier stages, this group is using sequential, low-magnification confocal imaging of fluorescently labeled cartilage and bone in live transgenic fish. At later stages, beyond the limit of confocal imaging, this project utilizes high-resolution microcomputed tomography (microCT) to generate detailed, 3D images of mineralized bones. The two methods are complementary; the confocal imaging offers a dynamic view of cell and tissue behavior and the ability to monitor changes in gene expression, whereas microCT is more amenable to larger sample numbers, quantitation, and statistical analysis. Importantly, the use of a novel contrast agent will improve the sensitivity of microCT at earlier stages, allowing the direct comparison of confocal images to mineralization in an individual fish. Capitalizing on the strength of genetic analysis in the zebrafish, the data on wild-type fish will be supplemented with similar analyses of selected mutants affecting the adult skull.

As an important extension of the description of transgene expression patterns, this group is developing transgenic lines to allow expression of different coding sequences in defined patterns, to serve a variety of purposes: fluorescent proteins for imaging; Cre recombinase for induced, tissue-specific genetic alterations (Kague et al., 2012); and normal or altered components of signaling pathways to manipulate them in specific cell types. Newly available tools for genome engineering will be used to alter selected endogenous loci appropriately, allowing insertion of cassettes containing different coding sequences. This approach ensures that expression of the inserted coding sequence accurately reflects endogenous gene expression. Through the combined generation of a comprehensive atlas and a set of transgenic and genetic tools, this project will substantially advance the use of zebrafish in the study of skull development and greatly facilitate comparative studies with mammals that will advance treatment options in human patients. This work will establish imaging approaches and genetic tools that can be used in the future to evaluate zebrafish models of human disorders, generated by these labs, by other projects in FaceBase, or by the wider community. Significantly, the spoke project described below is generating transcriptome atlases of the craniofacial sutures, which will complement the data from the zebrafish model and provide an opportunity for comparative analyses. This imaging data provides a baseline of normal skull development to compare with zebrafish mutants generated by other investigators, such as the ‘Rapid identification and validation of human craniofacial development genes’ project, and the imaging tools generated by this group can be used to help perform detailed phenotyping.

#### Transcriptome atlases of the craniofacial sutures

Craniofacial sutures are the fibrous joints between bones, allowing growth of the skull from prenatal to postnatal development until adult size is achieved. Craniosynostosis, or premature suture fusion, can restrict or alter skull growth and requires major surgical intervention to prevent secondary impairment to the brain, eyes, hearing, breathing, and mastication. It is a common birth defect, occurring in 1:2500 live births. Most mutations have been found in a small number of genes that account for the more common syndromic forms in humans (Heuze et al., 2014; Twigg and Wilkie, 2015). However, the underlying genetic etiology has not been identified for the majority of cases, which are typically nonsyndromic, single-suture synostoses. Craniofacial sutures also vary widely in form, function and susceptibility to fusion, suggesting that gene expression profiles vary among sutures. A basic distinction in gene expression can be seen between the non-ossifying suture mesenchyme and the flanking osteogenic fronts in cranial sutures, as exemplified by the expression of *Twist1* predominantly in suture mesenchyme and *Fgfr1-Fgfr3* in the osteogenic fronts and differentiating osteoblasts (Johnson et al., 2000). Other genes show suture-specific and developmental stage-specific expression (Rice et al., 2010; Holmes et al., 2015; Kyrylkova et al., 2015). Our understanding of suture biology and pathology would be aided greatly by a comprehensive knowledge of suture gene expression profiles. To this end, this project employs laser capture microdissection of 11 major craniofacial sutures at different embryonic stages, including embryonic day (E)16.5 and E18.5 in the wild type and craniosynostosis syndrome mouse models on the C57BL/6J background. The Apert syndrome *Fgfr2^+/S252W^* mouse model (Wang et al., 2005) will be analyzed for all 11 sutures, whereas the Saethre-Chotzen syndrome *Twist1^+/−^* mouse model (Chen and Behringer, 1995) will be analyzed for two sutures commonly affected in this model. RNA derived from the separated suture mesenchyme and osteogenic front sub-regions will be isolated to generate RNA-seq datasets. The final transcriptome atlases will comprise 635 individual datasets, including replicates. These will be delivered to the Hub at 6 month intervals for the duration of the project. These datasets will allow flexible strategies for the comparison of expression profiles within and across sutures representing a variety of cell lineages, anatomical locations, suture morphology, developmental time points and genotypes. This will accelerate both our understanding of human suture biology and the discovery of candidate genes whose mutation might cause craniosynostosis or other defects of craniofacial bone development. As they survey a wide variety of regions of active bone formation *in vivo*, these atlases will also be a key resource for uncovering general mechanisms of bone formation and pathology (facebase.org/projects/transcriptome-atlases-craniofacial-sutures/).

#### Genomic and transgenic resources for craniofacial enhancer studies

Genetic studies have shown that distant-acting regulatory sequences (enhancers) embedded in the vast non-coding portion of the human genome play important roles in craniofacial development and susceptibility to craniofacial birth defects. The mechanistic exploration of these distant-acting enhancers continues to be difficult because the genomic location and *in vivo* function of most craniofacial enhancers remains unknown (facebase.org/projects/craniofacial-enhancer-studies/).

**Fig. 2.**
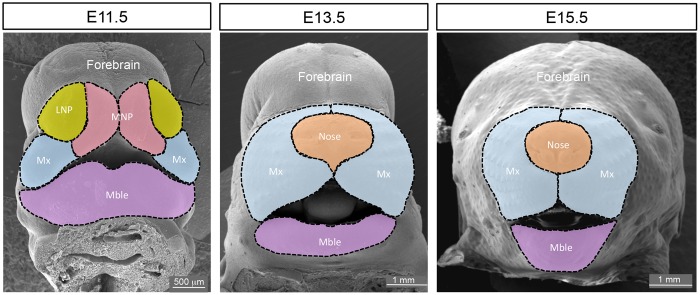
**Proposed sampling of mouse embryonic facial tissue for ChIP-seq.** LNP, lateral nasal process. MNP, medial nasal process. Mx, maxilla. Mble, mandible.

As members of FaceBase 1, this group generated the first genome-wide maps of enhancer-associated chromatin marks during craniofacial development, as well as enhancer reporter validation data for distal enhancers controlling craniofacial development in mice (Attanasio et al., 2013). These resources, including extensive optical projection tomography (OPT; Sharpe et al., 2002) data of enhancer activity patterns, proved to be of significant value to the craniofacial research community. However, these efforts captured only a small proportion of the enhancers that are active during craniofacial development *in vivo*. The goal of the current project is to characterize the gene regulatory landscape of craniofacial development more comprehensively using new and complementary approaches. ChIP-seq targeting active and repressive histone modifications is being performed on major mouse facial subregions at three embryonic time points that are critical for facial development (Fig. 2). The time points begin when the facial subregions are still distinct entities (E11.5) and continue through to when they fuse into an integrated whole and undergo differentiation into crucial components of the face, including cartilage, bone, teeth, muscle and exocrine glands (E13.5 and E15.5). This approach can greatly increase the number of identifiable enhancers active in mammalian tissues (Bonn et al., 2012). In addition, profiling a combination of characteristic active and repressive histone modifications produces complementary data types (Rada-Iglesias et al., 2011) that can be used to characterize enhancer activity *in vivo*. Preliminary data reveal tightly restricted temporal activity windows of developmental enhancers, consistent with observations in non-craniofacial tissues in the developing mouse (Nord et al., 2013). To complement this mouse-based effort, ChIP-seq on human embryonic face tissue will identify human-specific craniofacial enhancers that are not functionally conserved in mice. Integrated analysis of these data with RNA profiling of the same subregions (Fig. 2) will enable the identification of new regulatory networks and increase the understanding of previously identified interactions. In addition, this group supports other craniofacial research labs within and outside the FaceBase consortium by providing access to transgenic characterization resources as well as collaborative analysis of functional genomic and human genetic datasets related to craniofacial development and dysmorphologies. These projects include studies aimed at the identification of enhancers mechanistically involved in craniofacial etiologies such as cleft lip and palate (CLP), which is the most common birth defect in humans (Fakhouri et al., 2014; Gordon et al., 2014), and for regulating the development of the mandible and maxilla (see below).

#### Integrated research of functional genomics and craniofacial morphogenesis

Congenital malformations involving the facial bones significantly impact quality of life because our face is our identity. For example, mandibular dysmorphogenesis ranging from agenesis of the jaw to micrognathia is a common malformation and appears in multiple syndromes. Micrognathia not only presents as a facial deformity but can also cause cleft palate and airway obstruction, such as in Pierre Robin sequence (Parada et al., 2015). The maxilla contributes to mid-facial formation; maxillary hypoplasia is often associated with cleft palate and has been described in more than 60 different syndromes. Despite their importance, the mechanisms that regulate facial bone development are relatively uncharacterized (www.facebase.org/projects/functional-genomics-craniofacial-morphogenesis/).

Identification of anatomical landmarks and the average distances between them in control mice gives us the means to compare mutant models quantitatively. For example, data generated as part of FaceBase 1 led to the discovery that canonical and non-canonical TGF-β signaling have different relative involvement in the regulation of different craniofacial bones (Ho et al., 2015; Iwata et al., 2012, 2013, 2014). The depth and nuance of our understanding will increase as differences in gene expression in smaller domains, such as the proximal versus distal portions of the mandible, are compared. For this reason, the aim of the FaceBase 2 project is to investigate mandibular and maxillary development and malformations. MicroCT images of control and abnormally developing mice (including newly defined detailed anatomical landmarks and measurements), microarray gene expression datasets (including separate datasets for the proximal and distal regions of the mandible and maxilla for more detailed comparisons), and graphical representations of gene expression will be made available to the research community through the FaceBase Hub. Collectively, these data will fill a significant gap in our knowledge and generate invaluable resources for the research community. These data will be particularly enlightening when integrated with the related FaceBase-sponsored studies of enhancers, which may reveal how common regulatory genes exert their specific functions in mediating tissue-tissue interactions during craniofacial morphogenesis, as highlighted in the previous enhancer project. These datasets also have the potential to help researchers to generate new scientific questions. This work is a logical progression from this group's completed FaceBase 1 spoke project on palatal development, which generated 200 hard and soft tissue scans and 125 microarray gene expression datasets that are available from the FaceBase Hub. This microarray data will also be compared with the data generated in the project ‘RNA dynamics in the developing mouse face’ (see below). Many of these datasets are already benefitting the craniofacial research community and will continue to do so in the future.

#### RNA dynamics in the developing mouse face

Craniofacial morphogenesis is a complex process requiring coordinated proliferation, movement and differentiation of six distinct facial prominences. The complexity of this process leaves it vulnerable to environmental and genetic perturbations, such that craniofacial malformations are one of the most common classes of birth defects. Facial prominences are made up of a monolayer of ectoderm encasing a large core of mesenchymal cells derived from the neural crest and mesoderm. Signaling from this minor population of ectodermal cells directs and coordinates the behavior of the underlying mesenchyme, and thence facial morphogenesis. Manipulations that alter these signaling processes and tissue interactions have grave consequences for facial development, resulting in various types of medically important dysmorphology, including orofacial clefting. Thus, detailed knowledge of geno-dynamics of the ectoderm is an essential component of the overall description of facial development. A multi-disciplinary team has been assembled with expertise in craniofacial biology, mouse molecular genetics, bioinformatics and computational biology to gain a systems biology-level understanding of early mammalian facial development (facebase.org/projects/rna-dynamics-in-developing-mouse-face/).

The period between E10.5 and E12.5 of mouse embryogenesis represents the critical time from when the facial prominences first become distinct entities to when they fuse to form the external facial platform. During this developmental window, the distinct prominences that give rise to specific components of the face can be readily separated and analyzed to determine how their unique expression profiles correlate with their eventual fates. Such microdissection analyses are not possible after E12.5 because the prominences have merged and begun to form shared structures derived from several prominences. Therefore, this spoke will build on their previous analyses (Feng et al., 2009) by isolating the nasal, maxillary and mandibular prominences during these crucial stages of mouse facial development (E10.5, E11.5 and E12.5) and then separating the ectoderm and mesenchyme (Li and Williams, 2013). Steady-state levels of mature RNAs, including mRNAs and a variety of small RNAs, will be obtained from these separate tissues using RNA-seq and custom microarray approaches. The data will be analyzed using bioinformatic and molecular approaches to identify the various RNA isoforms in the developing face, as differential splicing is essential for face formation (Bebee et al., 2015). Lastly, miRNA targeting and translational potential will be studied to understand how these layers of RNA regulation are involved in differential gene expression. These combined studies should provide a valuable resource detailing the dynamic interplay of ectoderm and mesenchyme during normal facial development. Moreover, the datasets can be used to understand and interpret aspects of the E11.5 enhancer studies in the project ‘Genomic and transgenic resources for craniofacial enhancer studies’, as well as the refined positional information available from ‘Integrated research of functional genomics and craniofacial morphogenesis’.

## Data generated using humans or integrated human and animal models

### Epigenetic landscapes and regulatory divergence of human craniofacial traits

During development, cranial neural crest cells (CNCCs) play major roles in establishing craniofacial morphology and determining its species-specific variation. *In vivo*, human neural crest formation occurs at 3-6 weeks of gestation and is largely inaccessible to genetic and biochemical studies. To overcome the inability to obtain CNCCs directly from human and non-human higher primate embryos, this project employs a pluripotent stem-cell-based *in vitro* differentiation model in which specification, migration, and differentiation of CNCCs are recapitulated in the dish (Bajpai et al., 2010; Rada-Iglesias et al., 2012; Prescott et al., 2015). Using this *in vitro* model, genome-wide maps of select transcription factor and coactivator binding, histone modifications and chromatin accessibility will be generated for human CNCCs. Active cis-regulatory elements such as enhancers and promoters will be systematically annotated in these cells based on combinatorial chromatin signatures (Rada-Iglesias et al., 2011). This epigenomic profiling strategy will be further extended to the CNCCs from our nearest evolutionary cousin, the chimpanzee (Prescott et al., 2015). Given that cis-regulatory changes play a central role in morphological evolution, the project will catalogue the functional divergence of CNCC cis-regulatory elements between humans and chimpanzees, examine associated changes in gene expression and investigate mutations underlying recent human-chimp craniofacial evolution. Moreover, select enhancer elements that changed their regulatory activity during recent evolution will be analyzed *in vivo* in transgenic mouse embryos. This spoke will further interact with the FaceBase project led by Axel Visel (see above, ‘Genomic and transgenic resources for craniofacial enhancer studies’) to integrate and compare human and mouse craniofacial enhancer maps and will provide important data for cross-species comparison with the projects led by Yang Chai (‘Integrated research of functional genomics and craniofacial morphogenesis’) and Trevor Williams (‘RNA dynamics in the developing mouse face’).

### Rapid identification and validation of human craniofacial development genes

The advent of new genomic sequencing technologies has made the task of gene discovery in human developmental disorders highly efficient. Simultaneously, advances in gene targeting in model organisms such as zebrafish have made semi-high-throughput validation and analysis of human candidate genes feasible, including those responsible for craniofacial disorders (Coste et al., 2013; Faden et al., 2015; Gfrerer et al., 2014). This project takes advantage of this convergence of new technologies to identify and functionally validate ∼24 genes involved in novel aspects of human craniofacial development. Specifically, it utilizes already ascertained collections of craniofacial dysmorphoses from Boston Children's Hospital (BCH) and King Faisal Specialist Hospital and Research Center (KFSHRC) in Saudi Arabia, where the high incidence of consanguinity makes autozygosity mapping and the identification of recessive causal loci highly feasible. In autozygosity mapping, the inheritance of two copies of an ancestral allele as a result of consanguinity can reveal phenotypes caused by biallelic mutations in autosomal recessive genes and simultaneously facilitate the mapping of such mutations by flagging the surrounding haplotypes as tractable runs of homozygosity (ROH) (facebase.org/projects/rapid-id-validation-human-craniofacial-dev-genes/).

This project includes analysis of a relatively wide range of craniofacial disorders, including common cleft lip and palate, oblique facial clefts, hemifacial microsomia and other more uncommon anomalies in which gene basis remains to be elucidated. This group will specifically prioritize probands and family members with craniofacial dysmorphoses for whole exon sequencing (WES) who have: (1) diseases of unknown etiology consistent with *de novo* autosomal dominant inheritance; (2) multiple affected family members consistent with highly penetrant recessive inheritance; and (3) multiple affected family members in multiple generations, consistent with highly penetrant dominant inheritance. Depending on the individual pedigree, the team plans to sequence two to three individuals per case. In some cases, sequencing of the proband may be combined with less-expensive genotyping of other affected family members to determine genomic regions of identity by descent. Resources already compiled by FaceBase, including detailed gene expression data in mouse and zebrafish, enhancer analyses and genome-wide association studies, will further facilitate the functional annotation of these newly validated genes. The integration of available gene expression data from humans and model organisms (enriched by past and current FaceBase spoke projects) with the candidate gene list determined above can further improve the likelihood assessment that specific potential variants are causal for the disease phenotype. This project is employing zebrafish and mice in parallel as high-throughput platforms for initial screening of candidate gene expression and zebrafish for rapid functional analysis. The team hypothesizes that high-throughput *in vivo* spatiotemporal expression analysis and morpholino-mediated gene knockdown can rapidly provide provisional evidence for or against the causality of candidate gene variants implicated by WES or whole gene sequencing (WGS). Because morpholinos can yield non-specific effects, in all but the most clear-cut cases, this team will also prioritize selected, provisionally validated zebrafish genes that are homologous to human craniofacial candidate genes for TALEN or CRISPR-Cas gene targeting in zebrafish to generate germline mutants to further confirm the existence of a phenotype consistent with the human proband. The genomics pipeline is primed with cases within this project, but these investigators have also set up collaborations with other spoke projects within FaceBase 2 to recruit additional patients, such as with UCSF and USC. Data deposited in the FaceBase 2 Hub will include descriptions of patient phenotypes and clinical presentations, human candidate genes, animal gene expression data, and when available, phenotypic data from the animal model (zebrafish or mouse mutant). Scientists interested in craniofacial phenotypes will be able to examine the gene expression and phenotype data from the animal model. Human geneticists will be able to search for phenotypes of interest or syndromes and find potential candidate genes. The goal of this project is not only to build a resource to annotate craniofacial biology with candidate genes, but also to optimize the functional genomics technology that will facilitate this process and to position investigators at large to make use of these discoveries more rapidly to characterize the reported genes mechanistically.

### Developing 3D craniofacial morphometry data and tools to transform dysmorphology

Dysmorphology is the branch of pediatrics and clinical genetics concerned with structural birth defects and the delineation of syndromes. Thousands of syndromes that include craniofacial dysmorphology have been described. However, dysmorphology remains a largely descriptive art, with diagnoses based on subjective or semi-quantitative clinical impressions of facial and other anatomic features. This presents an important clinical limitation, as the inability to make precise diagnoses affects several aspects of patient care, including the ability to provide information regarding recurrence and prognoses, and can potentially impact management. Fortunately, over the past decade, dramatic technological advances in imaging, quantification and analysis of variation in complex 3D shape have revolutionized the assessment of morphologic variation, permitting robust definition of quantitative morphometric phenotypes that can distinguish patients from controls in a variety of syndromes (e.g. Goodwin et al., 2014; Manyama et al., 2014). The goal of this project is to build a library of 3D photographs of patients with a diverse array of craniofacial syndromes and to develop systems that will enable diagnostic application of craniofacial 3D morphometrics in clinical practice by defining specific quantitative measures that characterize the aberrant facial shapes in a large number of human dysmorphic syndromes. The long-term intent of this project is that 3D photomorphometric deep-phenotyping, in conjunction with the rapid advent of exome and genome sequencing in clinical medicine, will transform dysmorphology from a clinical art into a medical science. In addition, the library of photographs and the computational tools that are developed will provide an important resource for research in craniofacial biology (facebase.org/projects/3d-craniofacial-morph-transform-dysmorphology/).

## Computational tools to support craniofacial research

### The ontology of craniofacial development and malformation

The purpose of the FaceBase consortium is to acquire and integrate multiple forms of data systematically in order to facilitate a systems-level understanding of the causes and possible treatments for craniofacial abnormalities. A basic component of any such data integration effort is a controlled set of terms or keywords that can be associated with the data through data annotation, so that diverse data can be related via common terms. If in addition, the terms are related to each other in an ontology, then integration can occur at the level of meaning (semantics) rather than simply via keywords. For example, using relations such as ‘has malformation’, ‘has location’, ‘homologous to’ and ‘gives rise to’, the ontology could determine mouse developmental structures that give rise to tissues/organs homologous to human ones involved in craniofacial syndromes like Apert. A user could then simply ask for mouse expression data relevant to Apert, and a semantically based retrieval system could follow these relations to retrieve FaceBase mouse gene expression data annotated with the names of these mouse developmental structures. As part of FaceBase 1, this group designed and partially implemented the ontology of craniofacial development and malformation (OCDM), based on their foundational model of anatomy ontology (FMA). The FaceBase 1 version of the OCDM consisted of components for representing human and mouse adult and developmental anatomy and malformations, as well as mappings between homologous structures in the two organisms, with emphasis on those structures involved in cleft lip and palate (Brinkley et al., 2013; Mejino et al., 2013; Wang et al., 2014; Koul, 2015). In FaceBase 2, the OCDM is being enhanced to include conditions of interest to FaceBase 2 researchers, such as human, mouse and zebrafish facial, palatal and cranial vault development, and dysmorphology such as craniosynostosis, midface hypoplasia, frontonasal dysplasia, craniofacial microsomia and microtia (Mejino et al., 2015). Standardized terms from the OCDM and other ontologies are regularly supplied to the Hub for use in data annotation, which will later facilitate use of OCDM relations for semantic queries. OCDM content development uses existing terms and ontologies where available, and obtains and vets new terms in consultation with the Hub and other spoke projects. The OCDM is available through the FaceBase Hub and the OCDM project web page (Structural Informatics Group, 2015) (facebase.org/projects/ontology-of-craniofacial-development-and-malformation/).

## Human genomics analysis interface for FaceBase 2

There are now several large human genomics databases relevant to craniofacial research, including multiple databases funded in part by the first FaceBase consortium. Direct access to the individual-level data from such databases can be cumbersome; therefore, the current project seeks to make analysis of pertinent genomics data available to FaceBase users via a genomics analysis interface available through the FaceBase Hub without releasing the individual-level data. Craniofacial researchers may use this interface to mine these large datasets, for example if they have a comparable study, or if they have a gene of interest from expression or animal models (facebase.org/projects/human-genomics-analysis-interface/).

**Fig. 3.**
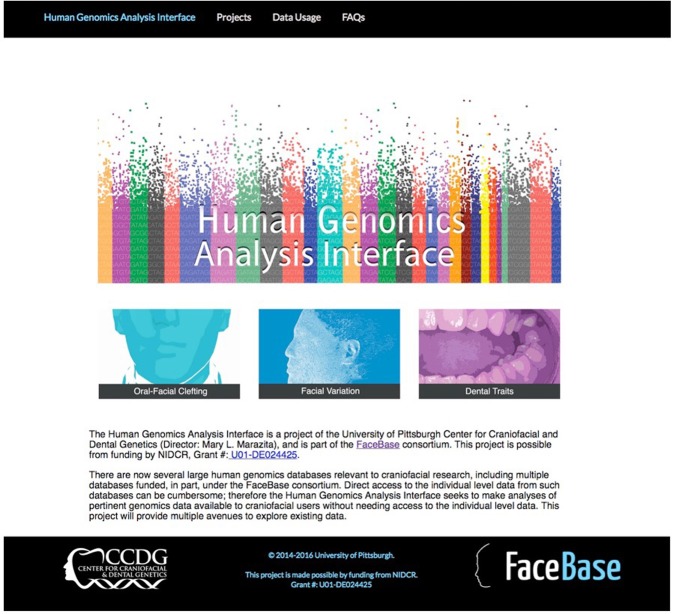
**Home page of the FaceBase Human Genomics Interface tool (facebase.sdmgenetics.pitt.edu), depicting the projects that are currently accessible through the tool.**

The interface provides: (1) summary data about each project including dbGaP accession number, relevant publications and descriptive statistics; (2) detailed pre-calculated results from statistical genetic analyses of the data from each project, for example, Manhattan plots for GWAS studies or Locus Zoom graphs; (3) the ability to request custom results by gene name, SNP name or genomic region; and (4) the ability to download PDFs of graphs or other materials generated. To date, the dbGaP Study projects available through the interface include: (1) ‘International Consortium to Identify Genes and Interactions Controlling Oral Clefts – Genome Wide Association Study’ (accession no.: phs000094.v1.p1; Beaty et al., 2010); (2) ‘Targeted Sequencing of CL/P GWAS Loci’ (phs000625.v1.p1; Leslie et al., 2015); (3) ‘GWAS of Orofacial Clefts in Guatemala’ (phs000440.v1.p1; Wolf et al., 2015). In preparation are interfaces to a large multi-ethnic orofacial cleft GWAS (PI, Mary Marazita) and to normal human facial variation projects: (1) ‘3D Facial Norms’ (FaceBase 1 project; Weinberg et al., 2016); (2) ‘Genetic Determinants of Orofacial Shape and Relationship to Cleft Lip/Palate’ (FaceBase 1 project; PI, Richard Spritz: phs000622.v1.p1). The interface is now available online at facebase.sdmgenetics.pitt.edu. Fig. 3 shows the home page of the interface.

## The FaceBase Hub: a community resource

**Fig. 4.**
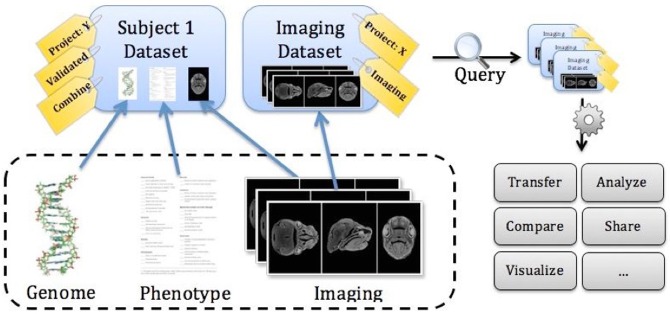
**An integrated dataset including imaging, genotypic and phenotypic data.** The Hub provides a faceted search infrastructure, allowing users to query the extensive metadata associated with each dataset. Data retrieved through queries can be transferred, compared, visualized, analyzed, shared, and more.

The breadth of spoke projects in the FaceBase consortium places unique requirements on data representation, management and use. Like many other research domains, craniofacial researchers typically work with large collections of files, often stored in different formats, spanning disparate locations and derived from different experiments. Seldom do researchers want to work with a single piece of data; rather they desire the ability to store, discover, move and analyze collections of data that relate to a particular operation. Associating these diverse data elements with one another and simplifying the task of working with integrated data can further enhance the breadth of exploration and make discovery possible. In support of these endeavors, the FaceBase 2 Hub provides tools to help researchers organize, manage and use data throughout its lifecycle. These tools enable users to organize heterogeneous and disparate data elements into problem-specific collections or datasets (Fig. 4). The FaceBase 2 Hub is built around the innovative concept of a biomedical digital asset management system (BDAM; Schuler et al., 2014) that was designed with the goal of making discovery and manipulation of complex scientific datasets as easy as it is to manage collections of photographs in commercial picture management software.

In order for the FaceBase Hub to serve the research community, it is crucial for researchers to be able to locate datasets of interest, conduct comparisons between datasets and understand how a particular dataset was derived. For example, researchers might want to assemble all datasets containing microarray data related to a given organism, project or study; locate a dataset used to derive a specific figure in a publication; check what stage of analysis has been completed on a given dataset; find all mouse models with a particular altered gene expression; or compare the imaging protocols used for a selected group of datasets. In FaceBase 2, we have created an innovative data discovery platform that enables researchers in the craniofacial research community to explore the different types of data available, interactively examine data in the Hub and download datasets of interest. A unified experimental data model based on the widely used ISA-TAB format (Sansone et al., 2012) leverages OCDM to provide structure to the data and to enable rapid discovery. An intuitive facet-based search model enables construction, bookmarking, and sharing of complex queries. Interactive data exploration tools, such as a 3D data viewer, enable users to explore data without leaving the Hub website, and linkages between FaceBase data and external repositories, such as the National Center for Biotechnology Information (NCBI) Gene Expression Omnibus (GEO) and the University of California Santa Cruz Genome Browser, enable users to explore connections between FaceBase data and other related sources. Finally, the Hub provides a RESTful web services API (Fielding and Taylor, 2002) enabling the integration of FaceBase data operations with external repositories and other tools.

**Fig. 5.**
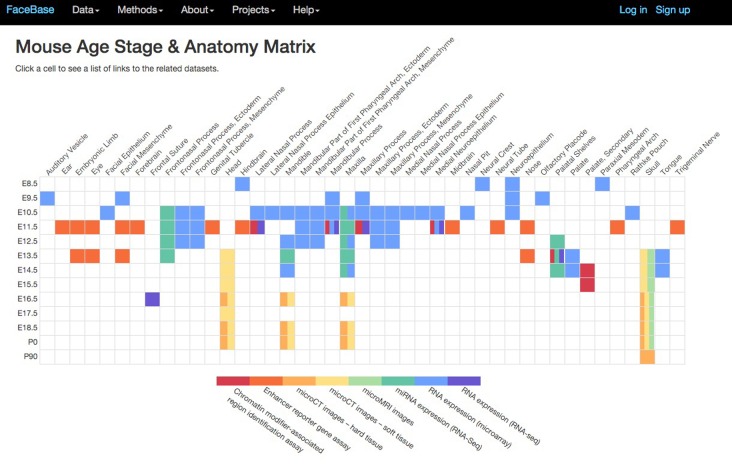
**Summary of mouse datasets available on the FaceBase Hub as of December 2015, organized by age stage and anatomical features.** Color-coding indicates the type of datasets available for the corresponding age stage and anatomical location. Clicking on any colored cell in the matrix leads to a list of links to the relevant datasets.

The genomics data generated by FaceBase 1 and 2 spoke projects are diverse and complex, spanning many different molecular measurements (from gene expression to genotyping to enhancer reporter assays) over a broad range of spatiotemporal points in diverse organisms. To take one example, Fig. 5 displays an overview of the currently available mouse datasets, arranged by anatomical feature and age stage and color-coded by data type. Clicking on any cell in this matrix leads to a list of all the relevant datasets. This matrix will grow as more mouse datasets come online; moreover, it represents only a small fraction of the Hub search capabilities, which allow for fully customizable queries over all the deposited FaceBase 1 and 2 datasets. New datasets are made accessible to the public via the Hub as soon as they are received. The Hub personnel have worked proactively with each spoke project to ensure support for submissions in all anticipated file formats, and continue to add tools and data structures progressively to support browsing and analysis of these data and integration across datasets.

For biologically intuitive data browsing of the extensive genomic data on craniofacial development in model organisms, a natural organization is by developmental time point and specific tissue or anatomical structure. For instance, there are 21 enhancer reporter experiments in FaceBase showing enhancer activity in the facial mesenchyme of the mouse at E11.5, and one RNA microarray showing global gene expression in the same tissue at E9.5. A user could take this list of enhancers and perform additional experiments (e.g. measure gene expression changes resulting from enhancer knockouts), specifically focusing on genes that are uniquely expressed in the facial mesenchyme. This list of uniquely expressed genes can be obtained by downloading the FaceBase global gene expression set for facial mesenchyme at E9.5 and comparing it with the FaceBase expression sets for other tissues at the same developmental stage. For expression datasets that have been curated by GEO, users can perform further analyses using the GEO data analysis tools.

Ultimately, the Hub will not merely serve as a collection point for the multifaceted data produced by the various spoke projects, but it will provide the means for integrating that data into a unified representation of what is known about craniofacial development. The set of new Hub browsing and navigation capabilities create new integrated craniofacial datasets that query all of the spoke data, collecting datasets by organism, developmental stage, phenotype, anatomical region of interest or gene being expressed. Hub personnel are also creating general navigation tools that link these data into interactive 3D and 2D image visualizations, enabling users to combine visual navigation with searches to explore linkages across FaceBase data. The OCDM vocabularies help drive these linkages. The goal is that these integrations will facilitate new analysis and expose genotype/phenotype connections that would otherwise not be apparent. In addition, the Hub is collaborating with the Monarch Initiative (monarchinitiative.org) and the developers of the Uberon (Mungall et al., 2012) to contextualize FaceBase data within a broader set of anatomical and phenotypic data. Linking FaceBase data with other data, such as that targeted toward other genetic disorders with phenotypes that may overlap those specifically associated with craniofacial dysmorphia, can reveal otherwise undetected genetic associations. We envision the Hub evolving into an essential research resource for a broader community as a consequence of the new types of connections that will only be possible within the FaceBase Hub.

## Perspectives

The FaceBase Consortium provides an important opportunity for the craniofacial community to come together for the advancement of our field as a whole. The joint efforts represented by the first and second rounds of FaceBase have prompted us to consider the advantages and challenges involved in such group science. First, the scientific community has the responsibility to promote data sharing and enhance the reproducibility of experimental results in the biomedical sciences (Collins and Tabak, 2014). By promoting a culture that emphasizes making data freely accessible, the FaceBase Consortium helps to satisfy the strong mandate to enforce transparency and integrity while serving as an accelerator for scientific discovery. The vast resources available through the FaceBase Hub will lay the groundwork for future hypothesis-driven research in the basic, translational and clinical sciences. These datasets will also serve as a rich resource for scientific collaborations, through which the seemingly divergent data sources can be utilized to advance scientific frontiers and provide innovative ways to study the regulatory mechanisms of craniofacial morphogenesis.

Second, given the ongoing parallel efforts in similar data consortia such as those studying the brain, kidney and lung, the National Institutes of Health have made a strong commitment to supporting concerted efforts to generate and distribute data resources for the research community. It will be crucial for various data hubs to communicate with each other to ensure a consistent and high-quality user experience across these different consortia. In parallel, each data consortium should begin to explore how to integrate existing datasets from the research community into the Hub in order to provide a broad and deep pool of resources for the research community.

Finally, it is important to reflect on how we measure the success of a consortium like FaceBase. FaceBase was created to generate and disseminate comprehensive data resources to empower the research community, to explore high-throughput technology, to foster new scientific collaborations among researchers and human/computer interactions, to facilitate hypothesis-driven research and to translate science into improved health care. These are challenging but achievable goals. Working together, we will accomplish them in service of the entire research community as well as the patients who can benefit most from our discoveries.
